# Differential expression of microRNAs in GH-secreting pituitary adenomas

**DOI:** 10.1186/1746-1596-5-79

**Published:** 2010-12-07

**Authors:** Zhi-Gang Mao, Dong-Sheng He, Jing Zhou, Bin Yao, Wei-Wei Xiao, Chun-Hua Chen, Yong-Hong Zhu, Hai-Jun Wang

**Affiliations:** 1 Department of Neurosurgery and Pituitary Tumor Center, The First Affiliated Hospital, Sun Yat-sen University, Guangzhou 510080, China; 2 Department of Histology and Embryology, Zhongshan School of Medicine, Sun Yat-sen University, Guangzhou 510080, China; 3 Department of Endocrinology and Pituitary Tumor Center, The First Affiliated Hospital, Sun Yat-sen University, Guangzhou 510080, China

## Abstract

**Background:**

The purpose of this study was to (1) identify specific miRNAs in growth hormones (GH)-secreting pituitary adenomas; (2) determine the relationship between the expression of these miRNAs and tumor size, somatostatin analogs treatment, and responsiveness to somatostatin analogs (SSA).

**Methods:**

Fifteen GH-secreting adenomas patients were treated with lanreotide for 4 months before surgery. Patients with 50% reduction of GH secretion by lanreotide were considered as SSA responders, while patients with less than 50% of GH reduction were considered as SSA nonresponders. We analyzed the miRNAs in 21 GH-secreting pituitary adenomas and 6 normal pituitaries by miRCURY™ LNA array and some differentially expressed miRNAs were validated by quantitative real-time PCR.

**Results:**

Fifty-two miRNAs were differentially expressed between GH-secreting pituitary adenomas and normal pituitaries. Differential expression of 9 miRNAs was observed between micro- and macro-adenomas. Thirteen miRNAs were differentially expressed between tumor samples from lanreotide-treated patients and those from lanreotide-untreated patients. Seven miRNAs were differentially expressed between SSA responders or GH nonresponders. Several identified miRNAs may be involved in cell proliferation, apoptosis, cancer development and progression.

**Conclusions:**

Our results indicate that altered miRNAs expression is involved in GH-secreting pituitary adenomas transformation, which will shed light on the mechanisms for the treatment of acromegaly by SSA. Identification and characterization of the targets of altered miRNAs genes may elucidate molecular mechanisms involved in the pathogenesis of pituitary adenoma.

## Background

MicroRNAs (miRNAs) are a class of non-coding RNAs that post-transcriptionally regulate the expression of downstream mRNAs by targeting the 3' untranslated regions [1,2]. Since the discovery that miRNAs are a class of conserved genes, hundreds of miRNA genes have been identified. More than 6000 miRNAs encoded by virus, plant and animal species have been recorded in miRBase bank [3,4]. miRNAs are a huge class of negative gene regulators controlling a wide range of biological functions such as cell proliferation, differentiation, signaling pathways, apoptosis and metabolism [5,6]. Recently, it has been shown that several human cancers, e.g. breast, colon, lung, brain, thyroid, and hematologic malignancies are associated with altered miRNAs expression [7]. In addition, more and more evidences suggested that some miRNAs might have oncogenic or tumor suppressor functions [8], and play an important role in tumorigenesis [9]. Previous studies have shown that expression of miR-15a and miR-16-1 in pituitary adenomas is lower than that in the normal pituitary tissues. Further more, the expression level of miR-15a and miR-16-1 is inversely correlated with tumor diameter and directly correlated with the secretion of the anti-neoplastic cytokine p43 [10]. Further studies have demonstrated that several identified miRNAs are involved in cell proliferation, apoptosis and corticotrophic tumorigenesis, suggesting that deregulation of miRNAs expression may be involved in pituitary tumorigenesis [11,12]. Predictive miRNAs could be potentially useful diagnostic markers, improving the classification of pituitary adenomas. Nevertheless, the role of transcriptional regulation of miRNAs and their target genes in the pathogenesis of pituitary adenomas remains largely unknown. Development of acromegaly is caused by the proliferation of somatotrophs and oversecretion of the hormone. A cascade of transcription factors and genetic elements normally determine the ability of somatotroph cells to synthesize and secrete growth hormone [13]. In this study, we compared the miRNAs expression between GH-secreting pituitary adenomas samples and normal pituitary samples by miRCURY™ Locked Nucleic Acid Array in order to identify miRNAs that are specifically associated with GH-secreting pituitary adenomas. The possible role of these identified miRNAs was also discussed.

## Materials and methods

### Patient information

The study was approved by the Institutional Review Board of the First Affiliated Hospital, Sun Yat-sen University (Guanzhou, China). The local ethical committee approved the pre-surgical medical treatment and all participants had informed written consent. The study is registered at ClinicalTrials.gov (NCT00993356). Tissue samples were collected in accordance with the guidelines of local committee on human research. The biological diagnosis of acromegaly was based on the criteria that (1) plasma GH concentration is higher than 1 μg/l after oral administration of 75 g of glucose (oral glucose tolerance test, OGTT); (2) insulin-like growth factor 1 (IGF-1) concentration is increased compared to the normal population in the same age and sex; and (3) relevant clinical features associated with acromegaly occurred and pituitary adenoma appeared on the magnetic resonance imaging examination.

### Experimental design

We examined 21 GH-secreting pituitary adenoma samples and found that 3 samples belonged to micro-adenomas (maximum diameter <10 mm), while the other 18 samples were macro-adenomas (maximum diameter >10 mm) [14]. Fifteen patients were treated with lanreotide (Somatuline Autogel, Beaufour Ipsen, Paris, France) for 4 months before surgery. Six patients did not receive any pre-surgical medical treatments. Previous conventional or stereotaxic (Gamma knife) radiotherapy was not performed for all the patients. Patients treated with lanreotide presented no evidence of previous cholecystolithiasis or any other abnormalities. As reported by Maiza, the initial dosage of lanreotide was 60 mg/28 days [15]. Patients with >50% reduction of GH secretion after treatment with lanreotide were considered as SSA responders, while patients with <50% GH secretion were considered as SSA nonresponders [16]. We assessed the responsiveness of GH secretion after the second injection because of the slow-release of lanreotide formulations. The dosage of lanreotide was adjusted according to the hormone response. In the case of nonresponders, the dosage of lanreotide was increased to 90 mg/28 days, and the dosage of lanreotide was maintained at 60 mg/28 days in the case of responders. SSA nonresponding patients received the treatment of lanreotide continuously because it ameliorated the clinical symptoms including headache, fatigue and peripheral soft-tissue swelling. All patients were well tolerated by lanreotide and they all completed the study. Twelve patients experienced bowel cramps and diarrhoea for several days after the first injection of lanreotide and the symptoms were resolved after the third injection. No adverse events were reported during the study. Magnetic resonance imaging scan of the pituitary, detection of plasma GH and IGF-1 concentration were performed after treatment.

### Detection of miRNAs expression and data analysis

Pituitary tumor samples were obtained during transphenoidal surgery. Tumor samples were divided two parts: one was used for pathologic analysis including hematoxylin eosin (H&E) and immunohistochemical staining and another was used for miRNAs expression. Tumor samples were snapped-frozen and stored at -80Ω°C. RNA isolation was performed according to the protocols provided by the manufacturer. Immunohistochemical staining for anterior pituitary hormones was performed on all samples to confirm hormone activity. Primary antibodies against the following antigens were used: prolactin (PRL, polyclonal, Code No A0569, 1:200, Dako), growth hormone (GH, polyclonal, Code No A0570, 1:400, Dako), follicle stimulating hormone (FSH, monoclonal, Code M3504, 1:50, Dako), luteinizing hormone (LH, monoclonal, Code M3502, 1:50, Dako), thyroid-stimulating hormone (TSH, monoclonal, Code M3503, 1:50, Dako), adrenocorticotropic hormone (ACTH, monoclonal, Code M3501, 1:50, Dako). In this study, all samples showed immunoreactivity of GH and negative for PRL, TSH, ACTH, FSH and LH. For miRNAs expression, tumor samples were micro-dissected by an experienced pathologist to remove any non-tumor tissues. As control, 6 normal pituitaries were obtained from the accidental death subjects during autopsies within six hours. There was no evidence of any previous endocrine diseases or other abnormalities in these accidental death subjects. All the sample tissues were disrupted by the Biopulverizer (biospec, Bartlesville, USA) and homogenized using the Mini-Bead-Beater-16 (biospec). Total RNAs were isolated by using TRIzol reagent (Invitrogen, Carlabad, CA) and RNeasy mini kit (Qiagen, Valencia, CA) according to the manufacturer's instruction. The quality and quantity of RNA were measured using a NanoDrop ND-1000 (NanoDrop). The ratio of OD_260_/_280 _for pure RNA should be close to 2.0 (ratios between 1.8 and 2.1 are acceptable). The ratio of OD_260_/_230 _should be larger than 1.8.

After the measurement of RNA quantity and quality, the samples are labeled using the miRCURY™ Hy3™/Hy5™ Power labeling kit (Exiqon, Denmark) according to the manufacturer's instruction. The Hy3™/Hy5™-labeled RNA molecules were hybridized on the miRCURY™ LNA Array (Exiqon, Denmark). The latest version of the array (v.10.0) consists of control probes, mismatched probes and more than 1200 capture probes, which cover all human, mouse and rat miRNAs sequences annotated in miRBase 10.0 at The Wellcome Trust Sanger Institute. Scanning was performed with the Axon GenePix 4000B microarray scanner. GenePix Pro 6.0 software (Axon Instruments, Union City, CA) was used to analyze the raw intensity of the images. Each sample was hybridized with miRCURY LNA™ Arrays in triplicate with three independent samples. The intensity of green signal was calculated after background subtraction and four replicated spots of each probe on the same slide were averaged. We used median normalization method to obtain "normalized data" based on the following formula: normalized data = (foreground-background)/median. The median is 50 percent quantile of the miRNA intensity, which was larger than 50 in all samples after background correction. After normalization, *student t*-test was used to identity the miRNAs that were differentially expressed between tumor and normal samples. Unsupervised hierarchical clustering and correlation analyses were performed for the miRNAs data. The differential expression of miRNAs was based on the criteria that (1) up- or down-regulated miRNAs were fold change >2.00 or <0.50, respectively; (2) the threshold value of false discovery rate (FDR) <0.10.

### Quantitative Real Time PCR

For quantitative real time PCR (qRT-PCR), total RNAs were extracted from pituitary adenomas with TRIzol reagent, digested with DNase I and reverse-transcribed into cDNA using applied biosystems 9700 Thermocycler (Applied Biosystems). The reverse transcription contained 1 μg of purified total RNAs, 2 μl of dNTP (2.5 mM each), 2 μl of RT Buffer (Epicentre), 1 μl of RT Prime (1 μM), 2 μl of MMLV reverse transcriptase and 0.3 μl of RNase inhibitor mix in a 20 μl volume. Reverse transcription was performed at 16°C for 30 min, 42°C for 30 min and 85°C for 5 min. All reverse transcription reactions were run in duplicate. qRT-PCR was performed using a standard TaqMan PCR kit protocol on the applied biosystems 7700 sequence detection system (Applied Biosystems). The 25 μl PCR included 2 μl of RT product, 1×TaqMan Universal PCR Master Mix (Applied Biosystems) according to the manufacturer's instruction. The reactions were incubated in a 96-well plate at 95°C for 5 min, followed by 40 cycles of 95°C for 10 sec, 60°C for 20 sec and 72°C for 20 sec. All reactions were run in triplicates for each data point, and data analysis was performed by using Sequence Detection System 1.9.1 software (Applied Biosystems). Primers used for qRT-PCR are listed in Additional file 1: Table S1. U6 was used as endogenous control to normalize the expression level of target genes because preliminary experiments showed that U6 expression level was very constant in all samples. Data was normalized by the endogenous control U6 Ct-median expression and calibrated by ^**Δ**^Ct-median value obtained from all sample tissues. Relative quantification of miRNA expression was calculated using the 2 ^-**ΔΔ**CT ^method [17].

We selected these miRNAs, such as miR-125a-5p, miR-125b, miR-524-5p etc, for qRT-PCR verification because these miRNAs were involved in the regulation of pituitary tumor-transforming gene (PTTG), insulin-like growth factor-binding protein 3 (IGFBP-3) and insulin-like growth factor-binding protein complex acid labile subunit chain precursor (IGFALS) based on the miRBase targets bank. We were particularly interested in the function of these miRNAs and their targets in the future research. Another reason that we selected these miRNAs for qRT-PCR was that some of these miRNAs were also reported to be differentially expressed in previous studies [11,12].

## Results

The diagnosis of GH-secreting pituitary adenoma was confirmed by H&E and immunohistochemical staining for all the samples (Additional file 2: Figure 1). In the 15 patients treated with lanreotide, 12 patients were considered as SSA responders and the remaining 3 patients were SSA nonresponders. The GH, IGF-1 level and the volume of tumors before and after lanreotide treatment were list in Additional file 1: Table S2. Significant shrinkage of the tumor volume was observed in two patients after treatment, we have published it in European Journal of Endocrinology [18].

**Figure 1:**
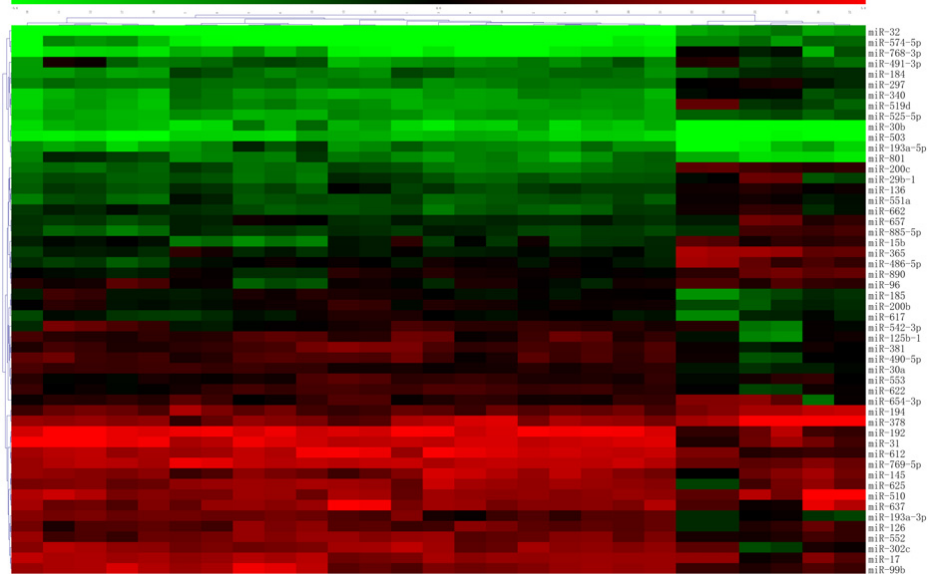
**Cluster analysis shows a clear distinction between GH-secreting pituitary adenomas and normal pituitary**. GH-secreting pituitary adenomas: 1-21, Normal pituitary: 22-27.

A total of 52 miRNAs were differentially expressed between GH-secreting pituitary adenomas and normal pituitaries. Twenty-three miRNAs were up-regulated and twenty-nine miRNAs were down-regulated in GH-secreting pituitary adenomas (Table 1). Although, the fold change of some up- and down-regulated miRNAs was >2.00 or <0.50, the miRNAs are not listed in Table 1 because the *P *value was >0.05 (miR-183, up, 2.73, *P *= 0.12, FDR = 0.021; miR-222, up, 2.87, *P *= 0.23, FDR = 0.023; miR-516b, up, 2.48, *P *= 0.16, FDR = 0.065; miR-601, up, 2.67, *P *= 0.11, FDR = 0.094; miR-629, up, 2.71, *P *= 0.31, FDR = 0.212; miR-886-5p, up, 3.49, *P *= 0.09, FDR = 0.025; miR-766, 2.53, *P *= 0.18, FDR = 0.033; miR-124, down, 0.29, *P *= 0.27, FDR = 0.005; miR-125a-5p, down, 0.41, *P *= 0.16, FDR = 0.029; miR-15a, down, 0.32, *P *= 0.21, FDR = 0.076; miR-198, down, 0.36, *P *= 0.25, FDR = 0.005; miR-630, down, 0.39, *P *= 0.26, FDR = 0.095; miR-744, down, 0.43, *P *= 0.24, FDR = 0.072; miR-765, down, 0.37, *P *= 0.14, FDR = 0.173). Cluster analysis showed a clear distinction between pituitary adenomas and normal pituitary (Figure 1). When adding the threshold value of FDR <0.10 to the differentially expressed criteria, eight miRNAs lost their status of differentially expressed (five in Table 1, two in Table 2 and one in Table 3).

**Table 1:** miRNAs differentially expressed in GH-secreting pituitary adenomas vs. normal pituitaries

Up regulated				Down regulated			
	
Name	Fold	*P *value	FDR	Name	Fold	*P *value	FDR
miR-136	3.52	0.005	0.005	miR-125b	0.16	0.035	0.033
miR-15b	4.29	0.021	0.062	miR-126	0.39	0.048	0.013
miR-184	2.17	0.033	0.033	miR-145	0.42	0.023	0.005
miR-194	2.13	0.047	0.029	miR-17	0.39	0.047	0.083
miR-200c	5.07	0.004	0.066	miR-185	0.25	0.012	0.023
miR-297	3.15	0.002	0.051	miR-192	0.12	0.005	0.046
miR-29b-1	2.39	0.042	0.016	miR-193a-3p	0.23	0.000	0.027
miR-32	5.48	0.030	0.020	miR-193a-5p	0.25	0.049	0.016
miR-340	5.69	0.004	0.066	miR-200b	0.41	0.002	0.061
miR-365	8.43	0.002	0.061	miR-302c	0.19	0.026	0.073
miR-378	3.79	0.046	0.077	miR-30a	0.43	0.000	0.027
miR-486-5p	4.28	0.043	0.043	miR-30b	0.41	0.004	0.046
miR-491-3p	2.56	0.041	0.025	miR-31	0.21	0.043	0.021
miR-519d	4.68	0.011	0.011	miR-381	0.45	0.003	0.066
miR-525-5p	2.57	0.009	0.072	miR-490-5p	0.36	0.032	0.042
miR-551a	3.31	0.012	0.183	miR-503	0.47	0.023	0.019
miR-574-5p	2.89	0.045	0.029	miR-510	0.21	0.046	0.081
miR-657	2.39	0.024	0.091	miR-542-3p	0.31	0.041	0.026
miR-662	3.21	0.045	0.017	miR-552	0.46	0.013	0.017
miR-768-3p	6.24	0.031	0.024	miR-553	0.33	0.031	0.198
miR-885-5p	2.66	0.015	0.053	miR-612	0.32	0.045	0.026
miR-890	2.93	0.036	0.302	miR-617	0.47	0.030	0.058
miR-96	2.38	0.031	0.082	miR-622	0.42	0.022	0.209
				miR-625	0.17	0.051	0.094
				miR-637	0.04	0.066	0.088
				miR-654-3p	0.38	0.030	0.180
				miR-769-5p	0.39	0.006	0.066
				miR-801	0.32	0.001	0.027
				miR-99b	0.38	0.003	0.061

Note: The threshold value used to screen up- and down-regulated miRNAs was fold change >2.00 or fold change <0.50.

**Table 2:** miRNAs differentially expressed with vs. without lanreotide treatment

Up regulated				Down regulated			
		
Name	Fold	*P *value	FDR	Name	Fold	*P *value	FDR
miR-183	2.47	0.043	0.021	miR-124	0.25	0.024	0.005
miR-193a-5p	2.82	0.026	0.025	miR-32	0.41	0.031	0.082
miR-222	2.96	0.035	0.049	miR-574-5p	0.42	0.041	0.073
miR-516b	2.31	0.033	0.065	miR-744	0.31	0.025	0.158
miR-524-5p	2.49	0.021	0.011	miR-96	0.30	0.017	0.051
miR-601	2.58	0.030	0.094				
miR-629	2.85	0.050	0.212				
miR-99b	2.29	0.032	0.034				

Note: The threshold value used to screen up- and down-regulated miRNAs was fold change >2.00 or fold change <0.50.

**Table 3:** miRNAs differentially expressed in macro- vs. micro- GH-secreting pituitary adenomas

Up regulated				Down regulated			
	
Name	Fold	*P *value	FDR	Name	Fold	*P *value	FDR
miR-184	2.68	0.043	0.029	miR-124	0.43	0.031	0.005
miR-524-5p	2.85	0.021	0.027	miR-222	0.36	0.022	0.023
miR-629	2.49	0.015	0.061	miR-32	0.47	0.011	0.016
miR-766	2.17	0.031	0.033	miR-744	0.42	0.020	0.072
				miR-765	0.41	0.042	0.173

Note: The threshold value used to screen up- and down-regulated miRNAs was fold change >2.00 or fold change <0.50.

**Figure 2:**
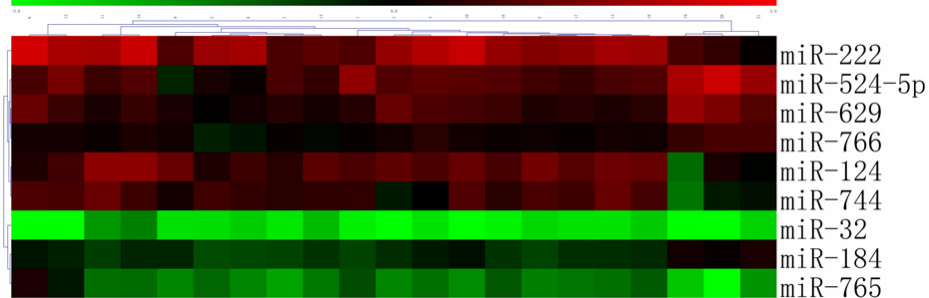
**Cluster analysis shows a clear distinction between macro- and microadenomas in GH-secreting pituitary adenomas**. Macroadenomas:1-18, Microadenomas:19-21.

**Figure 3:**
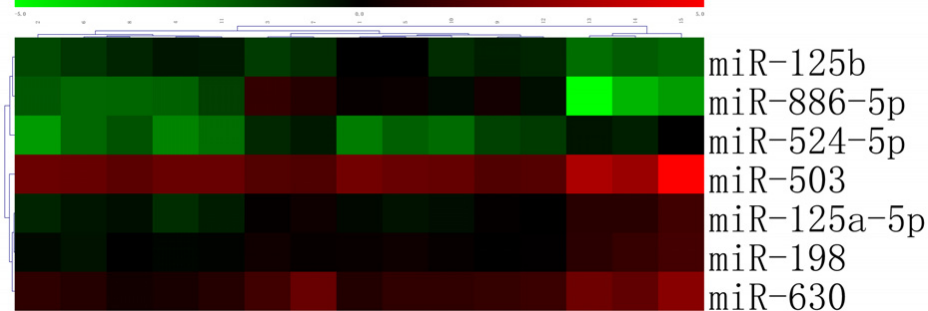
**Cluster analysis shows a clear distinction between SSA Responders and SSA Noresponders in GH-secreting pituitary adenomas**. SSA Responders:1-12, SSA Noresponders:13-15.

A total of 9 miRNAs were differentially expressed between macro- and micro-adenomas (Table 3). Cluster analysis based on these differentially expressed miRNAs showed that macro- and micro-adenomas belonged to two distinct groups (Figure 2). A total of 13 miRNAs were differentially expressed between the GH-secreting pituitary adenomas with lanreotide-treated patients and those without lanreotide-treatment (Table 2). We also found that 7 miRNAs were differentially expressed between SSA responders and SSA nonresponders (Table 4). Cluster analysis based on these differentially expressed miRNAs showed a clear distinction between SSA responders and SSA nonresponders (Figure 3).

**Table 4:** miRNAs differentially expressed in treatment with GH responders vs. GH noresponders

Up regulated				Down regulated			
	
Name	Fold	*P *value	FDR	Name	Fold	*P *value	FDR
miR-125b	2.73	0.034	0.013	miR-125a-5p	0.42	0.043	0.029
miR-886-5p	12.41	0.015	0.025	miR-198	0.46	0.012	0.005
				miR-503	0.31	0.031	0.077
				miR-524-5p	0.41	0.036	0.033
				miR-630	0.42	0.022	0.095

Note: The threshold value used to screen up- and down-regulated miRNAs was fold change >2.00 or fold change <0.50.

To verify the results obtained by microarray analysis, we performed qRT-PCR for some of the differentially expressed miRNAs. qRT-PCR results for the expression of miR-124, miR-125a, miR-126, miR-223, miR-381, miR-503, miR-524-5p, miR-525-5p, and miR-886-5p were consistent with the results obtained from microarray analysis (Table 5). In supplementary experiments, we performed qRT-PCR for analyzing more miRNAs expression (Table 5). As described by Bottoni [11], prediction of miRNAs target genes can be analyzed using four algorithms: TargetScan, PicTar, miRanda and miRBase Targets http://microrna.sanger.ac.uk/targets/. Using these algorithms, we performed prediction analysis for the target genes of the differentially expressed miRNAs identified in microarray (miR-124, miR-125a-5p, miR-125b, miR-126, miR-145, miR-151-3p, miR-524-5p, miR-516b, miR-744 and miR-96). We also analyzed the target genes of qRT-PCR confirmed miRNAs (miR-223, miR-381, miR-503, miR-525-5p and miR-886-5p).

**Table 5:** Some microarray data validation by qRT-PCR

Different miRNAs			U6	
	
	Microarray	Real time	Microarray	Real time
miR-124	0.54	0.39	1	1
miR-125a	1.26	1.95	1	1
miR-126	0.39	0.42	1	1
miR-223	2.78	1.42	1	1
miR-381	0.86	0.96	1	1
miR-503	0.22	0.37	1	1
miR-524-5P	0.28	0.34	1	1
miR-525-5p	5.14	4.05	1	1
miR-886-5P	3.71	1.51	1	1
miR-125b	2.85	1.92	1	1
miR-145	1.60	1.76	1	1
miR-151-3p	1.16	0.91	1	1
miR-183	1.20	1.59	1	1
miR-184	0.63	0.77	1	1
miR-193a-5p	0.62	0.73	1	1
miR-194	1.11	0.84	1	1
miR-198	0.46	0.49	1	1
miR-222	1.12	1.42	1	1
miR-30b	0.49	1.11	1	1
miR-32	1.64	1.03	1	1
miR-516b	0.64	0.87	1	1
miR-574-5p	1.10	1.15	1	1
miR-601	0.53	0.79	1	1
miR-629	0.45	0.31	1	1
miR-630	0.38	0.48	1	1
miR-744	2.03	1.74	1	1
miR-765	1.49	2.11	1	1
miR-766	0.57	0.47	1	1
miR-96	1.85	2.23	1	1
miR-99b	2.53	1.19	1	1

Note: Relative miRNA mean levels in pituitary adenomas were evaluated by quantitative real time PCR in three individual experiments.

## Discussion

### GH-secreting pituitary adenoma and normal pituitary

Among the differentially expressed miRNAs between GH-secreting pituitary adenomas and normal pituitary samples, some are involved in cell growth, apoptosis, cell proliferation and tumor development, as reported by the previous studies [19-21]. Yu et al. [19] found that ectopic overexpression of miR-184 resulted in a marked increase in apoptosis and cell death. Meanwhile, Hino et al. [20] demonstrated that miR-194 was involved in the differentiation of intestinal epithelial cells. Ferretti et al. [21] found that down-regulation of miR-125b caused the proliferation of tumor cells. In our study, we found that miR-126 and miR-381 were down-regulated in GH-secreting pituitary adenomas compared to normal pituitary. Previous study has shown that miR-126 is located in the chromosome region of 9q34.3 and regulates phosphatidylinositol 3-kinase (PI3K) signaling by targeting the PI3K regulatory subunit beta (p85b). Guo et al. [22] reported that miR-126 modulated the activity of PI3K at the level of signal initiation by limiting p85b levels in the normal colon epithelium. Loss of miR-126 during tumorigenesis would eliminate this check point and facilitate the amplification of PI3K signal, which may provide a growth advantage during colon carcinogenesis. The target of miR-126 and miR-381 is PTTG protein 1, which is involved in multiple cellular pathways, including proliferation, DNA repair, transformation, angiogenesis induction, invasion, and the induction of genetic instability. PTTG is overexpressed in most pituitary adenomas and is correlated to the recurrence and angiogenesis [23]. Our results, therefore, indicated that altered expression of miR-126 genes may play an important role in the development of GH-secreting pituitary adenomas.

### Tumor size

Among 9 differentially expressed miRNAs between micro- and macro- GH-secreting pituitary adenomas, the expression of miR-15a was down-regulated. There was no correlation between the reduced expression of miR-15a and tumor size (miR-15a, down-regulated, 0.32, *P *= 0.21, FDR = 0.076, A *P *value >0.05, the data wasn't shown in the Table 1). This finding was supported by previous study using the samples of corticotropinoma [12]. Bottoni et al. [10] demonstrated an inverse correlation between the tumor diameter and the expression level of miR-15a and miR-16 in samples of GH- or PRL-secreting pituitary adenomas, which was different from our findings. In short, these findings suggested a role of reduced expression of miR-15a and miR-16 in the pathogenesis of pituitary tumors. Consistent with these results, enforced expression of the miR-222 can induce the thyroid papillary carcinoma cell line to progress to the S phase of the cell cycle, indicating that miR-222 negatively regulates p27Kip1 protein expression and cell cycle [24]. Amaral et al. [12] found that the expression of miR-21, miR-141 and miR-150 was reduced in corticotropinomas. Patients with lower expression of miR-141 had a higher chance of remission after transphenoidal surgery, suggesting a possible role of the miR-141 in the regulation of pituitary genes involved in tumor growth and tumor local invasion. However, in this study, we did not observe significant difference of miR-21 and miR-141 expression between micro- and macro-GH-secreting pituitary adenomas. Further studies are needed to elucidate the pathogenesis of different subtype pituitary tumors including GH-secreting pituitary adenomas, PRL-secreting pituitary adenomas, and corticotropinoma.

### Lanreotide treatment and SSA responders or nonresponders

Among the putative targets of miRNAs, miR-125a-5p, miR-125b and miR-524-5p are associated with IGFBP-3 and IGFALS chain precursor, which are involved in protein binding, receptor binding, cell communication and regulation of growth. The role of IGFALS is associated with the regulation of the bioavailability of IGFs during postnatal growth. Up to 90% of circulating IGF-I and IGF-II are carried by binding to either IGFBP-3 or IGFBP-5. ALS, in the form of tertiary complexes, can extend their circulating half-life of IGF-I and IGF-II [25]. Moreover, miR-524-5p targets matrix metalloproteinase-9, which is involved in metabolism, ion binding and extracellular matrix. However, miR-524-5p was down-regulated in SSA responders as compared to SSA nonresponders. In contrast, miR-524-5p was up-regulated in the lanreotide-treated patients as compared to the untreated patients. The possible reason of miR-524-5p up and down regulation in different groups is due to the different responsiveness to SSA treatment in various cases. The response to SSA depends on the presence of a sufficiently high number of somatostatin receptors (SSRs) (subtypes, sst1-5) on the tumor cells. GH secretion is regulated through ligand binding of somatostatin to both sst2 and sst5, whereas SSA binds preferentially sst2 [26]. The GH-lowering effect of SSA is positively correlated with the level of sst2 mRNA expression [27-29]. Thus, in GH-responder cases, the level of growth factor is high. Lanreotide is a type of inhibitory growth factor. It is possible that miR-524-5p is negatively correlated with growth factors, e.g., lanreotide promotes miR-524-5p up regulation and inhibits the growth factors. Another possible reason is that miR-524-5p is inhibited itself by growth factor. When the inhibition is removed (lanreotide treatment), the level of expression of miR-524-5p is up-regulated. The similar reason may explain the opposite phenomenon occurred with the miR-193a-5p, miR-574-5p, miR-96 and miR-99b in Table 2 and Table 5. However, further studies are needed to elucidate the function and mechanisms of altered expression of miR-524-5p.

Further analyses showed that miR-516b and miR-96 target IGFBP-7, miR-744 targets IGFBP-6, and miR-99b targets homeobox protein prophet of Pit-1. All these target genes are involved in the regulation of organ development, nucleic acid binding and membrane-bound organelles. Visvanathanet et al. [30] demonstrated that SCP1 (small C-terminal domain phosphatase 1) played an anti-neural role during CNS development. miR-124 can inhibit SCP1 expression by directly targeting SCP1-3' untranslated region (UTR). It is suggested that during CNS development, timely down-regulation of SCP1 is critical for induction of neurogenesis. Contribution of miR-124 to this process is at least partially through down-regulation of SCP1 expression. These results also implied that establishing a novel evolutionarily conserved strategy to keep the balance between miRNAs and their transcriptional regulatory programs is necessary.

miR-145 was down-regulated in GH-secreting pituitary adenomas as compared to normal pituitary. Amaral et al. [12] observed that miR-145 was down-regulated in 11 samples of corticotropinomas, suggesting a possible role of miR-145 in carcinogenesis. The potential target genes of miR-145 encode oncogenic proteins, such as *myc, kras, fos, yes*, *fli*, cyclin D2, and MAPK transduction proteins [31]. miR-145 targets the insulin receptor substrate-1 (IRS-1) and miR-151-3p targets the insulin receptor substrate-4 (IRS-4), which regulated cell communication, receptor and membrane activities. Furthermore, Shi et al. [32] demonstrated experimentally that miR145 targeted IRS-1 and had a profound biological effect on human colon cancer cells. IRS-1, a docking protein for both the type 1 insulin-like growth factor receptor (IGF-IR) and the insulin receptor, is known to transmit a mitogenic, anti-apoptotic, and anti-differentiation signal.

Some of the miRNAs in our study, such as miR-769-5p, miR-885-5p, miR-886-5p and miR-890 are newly discovered in pituitary adenomas samples because the new array contains more capture probes and their functions are unknown [33]. The differentially expressed miRNAs are correlated with adenoma characteristics. In this study, the sample size is relatively low, and 4 months of treatment is relatively short. However, it must be emphasized that our studies represent promising preliminary results. Therefore, further studies are needed to predict up-regulated or down-regulated miRNAs target genes and their correlation with GH-secreting pituitary adenomas characteristics.

In conclusion, our results indicated that altered miRNAs expression may be involved in GH-secreting pituitary adenomas transformation. Furthermore, some differentially expressed miRNAs are associated with tumor diameter, lanreotide treatment, and responsiveness to SSA. These results will facilitate our understanding on mechanism of SSA treatment for acromegaly. Further studies are needed to predict the up-regulated or down-regulated miRNAs' targets and their partner factors in pituitary adenomas. Studying the targets of deregulated miRNAs may elucidate molecular mechanisms involved in pituitary adenoma pathogenesis.

## Competing interests

The authors declare that they have no competing interests.

## Authors' contributions

ZGM carried out the research studies, participated in the drafting of the manuscript. DSH and JZ carried out the immunohistochemical studies and detection of miRNAs expression and data analysis. BY participated in the design of the study. YHZ and HJW conceived of the study, helped draft the manuscript and participated in its design and coordination. All authors read and approved the final manuscript.

## Supplementary Material

Additional file 1 **Table S1 and S2**. Supplemental tables Click here for file

Additional file 2 **Figure S1. Diagnosis of GH-secreting pituitary adenoma with H&E and immunohistochemical staining**. Panel A indicated the microphotographs with H&E staining for growth hormone pituitary adenoma sections. A strong and diffuse acidophilic staining was shown in the cytoplasm of tumor cells. (original magnification, 200×). Panel B indicated microphotographs of immunohistochemically stained tumor sections, the tumor shows strong and diffuse staining for growth hormone antigen (original magnification, 400×). Click here for file
