# Supplementary material for: Differential expression of microRNAs in GH-secreting pituitary adenomas
Source: Diagn Pathol. 2010 Dec 7;5:79. doi: 10.1186/1746-1596-5-79 (PMC3017030; doi:10.1186/1746-1596-5-79)
Supplement: Additional file 1 — Table S1 and S2. Supplemental tables [file 1746-1596-5-79-S1.DOC]

Additional files

**Supplemental** table 1. Sequence of primers for qRT-PCR

| Gene name | RT primer |
| --- | --- |
| U6 | 5’CGCTTCACGAATTTGCGTGTCAT3’ |
| miR-124 | 5’ UAAGGCACGCGGUGAAUGCC3’ |
| miR-125a-5p | 5’ UCCCUGAGACCCUUUAACCUGUGA3’ |
| miR-126 | 5’ UCGUACCGUGAGUAAUAAUGCG3’ |
| miR-223 | 5’ UGUCAGUUUGUCAAAUACCCCA3’ |
| miR-381 | 5’ UAUACAAGGGCAAGCUCUCUGU3’ |
| mir-503 | 5’ UAGCAGCGGGAACAGUUCUGCAG3’ |
| miR-524-5p | 5’ GAAGGCGCUUCCCUUUGGAGU3’ |
| miR-525-5p | 5’ CUCCAGAGGGAUGCACUUUCU3’ |
| miR-886-5p | 5' CGGGUCGGAGUUAGCUCAAGCGG3' |
| miR-125b | 5' UCCCUGAGACCCUAACUUGUGA3' |
| miR-145 | 5' GUCCAGUUUUCCCAGGAAUCCCU3' |
| miR-151-3p | 5'CUAGACUGAAGCUCCUUGAGG 3' |
| miR-183 | 5'UAUGGCACUGGUAGAAUUCACU 3' |
| miR-184 | 5' UGGACGGAGAACUGAUAAGGGU3' |
| miR-193a-5p | 5' UGGGUCUUUGCGGGCGAGAUGA3' |
| miR-194 | 5' UGUAACAGCAACUCCAUGUGGA3' |
| miR-198 | 5' GGUCCAGAGGGGAGAUAGGUUC3' |
| miR-222 | 5' AGCUACAUCUGGCUACUGGGU3' |
| miR-30b | 5' UGUAAACAUCCUACACUCAGCU3' |
| miR-32 | 5' UAUUGCACAUUACUAAGUUGCA3' |
| miR-516b | 5' AUCUGGAGGUAAGAAGCACUUU3' |
| miR-574-5p | 5'UGAGUGUGUGUGUGUGAGUGUGU 3' |
| miR-601 | 5'UGGUCUAGGAUUGUUGGAGGAG 3' |
| miR-629 | 5' UGGGUUUACGUUGGGAGAACU3' |
| miR-630 | 5'AGUAUUCUGUACCAGGGAAGGU 3' |
| miR-744 | 5' UGCGGGGCUAGGGCUAACAGCA3' |
| miR-765 | 5' UGGAGGAGAAGGAAGGUGAUG3' |
| miR-766 | 5' ACUCCAGCCCCACAGCCUCAGC3' |
| miR-96 | 5' UUUGGCACUAGCACAUUUUUGCU3' |
| miR-99b | 5' CAAGCUCGCUUCUAUGGGUCUG3' |

**Supplemental** table 2. Efficacy of lanreotide therapy for acromegaly

| Patient no. | Age (yr) | sex | | GH Levels (μg/L) | | | IGF-1 Levels (μg/L) | | | Tumors Size(mm3) | |
| --- | --- | --- | --- | --- | --- | --- | --- | --- | --- | --- | --- |
|  |  |  |  | Pretherapy | On Therapy | | Pretherapy | On Therapy | | Pretherapy | On Therapy |
| 1 | 38 | M | 35.2 | | 9.42 | 483 | | 325 | 5529 | | 3518 |
| 2 | 40 | M | 12.6 | | 11.8 | 467 | | 386 | 28852 | | 20195 |
| 3 | 46 | F | 65.2 | | 38.0 | 542 | | 371 | 6623 | | 5058 |
| 4 | 54 | M | 67.4 | | 9.3 | 719 | | 406 | 4768 | | 3015 |
| 5 | 38 | F | 17.2 | | 2.2 | 536 | | 334 | 15394 | | 11816 |
| 6 | 31 | M | 161.3 | | 65.9 | 661.6 | | 501 | 31993 | | 27023 |
| 7 | 39 | M | 78.1 | | 24.1 | 415 | | 343 | 9236 | | 6283 |
| 8 | 32 | F | 59.2 | | 15.0 | 1134 | | 782 | 6963 | | 3979 |
| 9 | 43 | M | 63.2 | | 5.99 | 512 | | 382 | 7779 | | 4775 |
| 10 | 30 | F | 35.1 | | 3.56 | 791 | | 460 | 31993 | | 16336 |
| 11 | 37 | M | 123.7 | | 2.98 | 588 | | 539 | 15247 | | 9247 |
| 12 | 32 | F | 63.2 | | 7.45 | 697 | | 386 | 8092 | | 4948 |
| 13 | 27 | F | 38.3 | | 3.44 | 421 | | 243 | 17417 | | 10681 |
| 14 | 29 | F | 89.1 | | 6.23 | 857 | | 540 | 46108 | | 20525 |
| 15 | 26 | M | 76.5 | | 3.76 | 1058 | | 834 | 9414 | | 1851 |
