# Supplementary figures and images for: Differential expression of microRNAs in GH-secreting pituitary adenomas
Source: Diagn Pathol. 2010 Dec 7;5:79. doi: 10.1186/1746-1596-5-79 (PMC3017030; doi:10.1186/1746-1596-5-79)

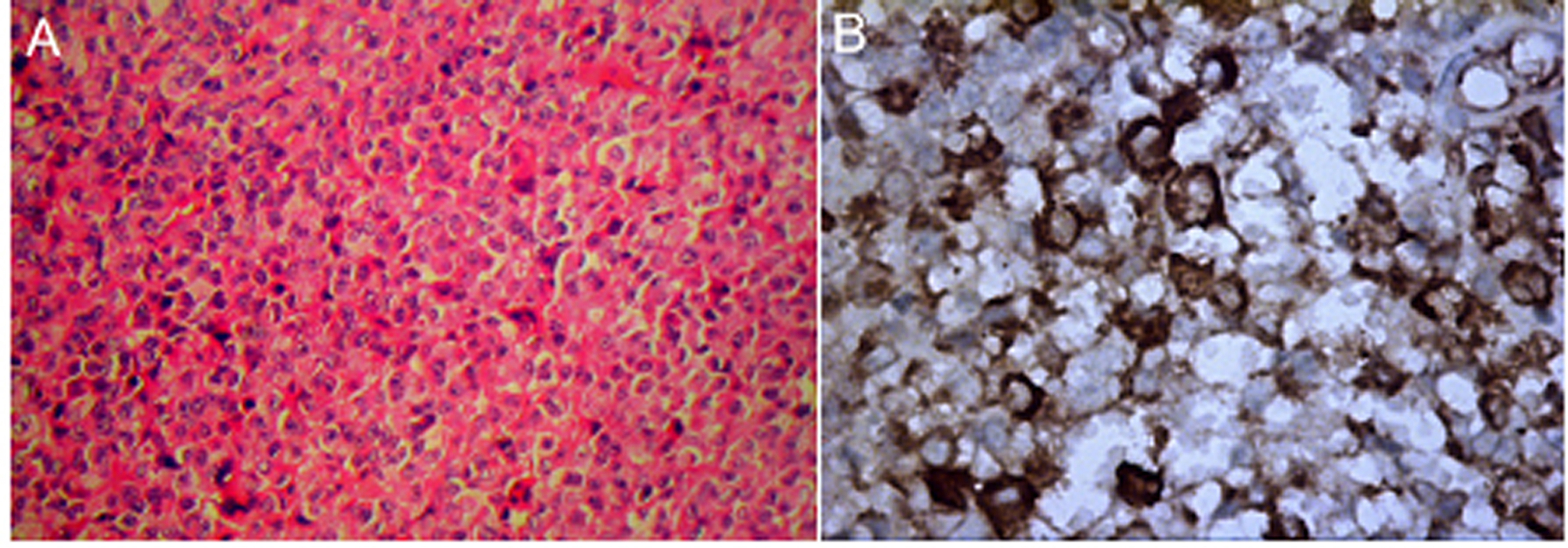

Supplement: Additional file 2 — Figure S1. Diagnosis of GH-secreting pituitary adenoma with H&E and immunohistochemical staining. Panel A indicated the microphotographs with H&E staining for growth hormone pituitary adenoma sections. A strong and diffuse acidophilic staining was shown in the cytoplasm of tumor cells. (original magnification, 200×). Panel B indicated microphotographs of immunohistochemically stained tumor sections, the tumor shows strong and diffuse staining for growth hormone antigen (original magnification, 400×). [file 1746-1596-5-79-S2.JPEG]
